# Machining learning predicts the need for escalated care and mortality in COVID-19 patients from clinical variables

**DOI:** 10.7150/ijms.51235

**Published:** 2021-02-18

**Authors:** Wei Hou, Zirun Zhao, Anne Chen, Haifang Li, Tim Q. Duong

**Affiliations:** 1 Department of Family, Population and Preventive Medicine, Renaissance School of Medicine at Stony Brook University, Stony Brook, New York, United States of America.; 2 Department of Radiology, Renaissance School of Medicine at Stony Brook University, Stony Brook, New York, United States of America.; 3 Department of Radiology, Montefiore Medical Center and Albert Einstein College of Medicine, Bronx, New York, United States of America.

**Keywords:** coronavirus 2 (SARS-CoV-2), pneumonia, artificial intelligence, lung infection

## Abstract

**Objective:** This study aimed to develop a machine learning algorithm to identify key clinical measures to triage patients more effectively to general admission versus intensive care unit (ICU) admission and to predict mortality in COVID-19 pandemic.

**Materials and methods:** This retrospective study consisted of 1874 persons-under-investigation for COVID-19 between February 7, 2020, and May 27, 2020 at Stony Brook University Hospital, New York. Two primary outcomes were ICU admission and mortality compared to COVID-19 positive patients in general hospital admission. Demographic, vitals, symptoms, imaging findings, comorbidities, and laboratory tests at presentation were collected. Predictions of mortality and ICU admission were made using machine learning with 80% training and 20% testing. Performance was evaluated using receiver operating characteristic (ROC) area under the curve (AUC).

**Results:** A total of 635 patients were included in the analysis (age 60±11, 40.2% female). The top 6 mortality predictors were age, procalcitonin, C-creative protein, lactate dehydrogenase, D-dimer and lymphocytes. The top 6 ICU admission predictors are procalcitonin, lactate dehydrogenase, C-creative protein, pulse oxygen saturation, temperature and ferritin. The best machine learning algorithms predicted mortality with 89% AUC and ICU admission with 79% AUC.

**Conclusion:** This study identifies key independent clinical parameters that predict ICU admission and mortality associated with COVID-19 infection. The predictive model is practical, readily enhanced and retrained using additional data. This approach has immediate translation and may prove useful for frontline physicians in clinical decision making under time-sensitive and resource-constrained environment.

## Introduction

Coronavirus Disease 2019 (COVID-19) 1-3, first reported in Wuhan, China in December 2019, was declared a pandemic on March 11, 2020 by the World Health Organization 4. As of June 26, 2020, COVID-19 has already infected 10 million and killed over 500,000 individuals worldwide 5. The actual numbers are likely to be much higher due to testing kit shortages and potential under-reporting. The sudden outbreak and rapid spread of COVID-19 have strained hospital resources, such as personal protective equipment, intensive care unit beds and mechanical ventilators. There will likely be a second wave and recurrence 6. There are currently no established prognostic biomarkers to accurately predict which patients are at imminent risk of death or require immediate escalated care, making resource allocation difficult. This challenge is further magnified by the large number of clinical lab markers being affected by COVID-19 infection (see reviews 7-9), the incompletely understood disease course, and the heterogeneous presentations. For example, some patients have mild or asymptomatic infections, while others exhibit severe symptoms. Some patients exhibit a mild disease course, while others deteriorate rapidly with multi-organ failure. Together, these challenges underscore the need to effectively triage and manage the care of COVID-19 patients, particularly in resource-constrained environments. There is currently no consensus as to which clinical variables are predictive of mortality and the needs for escalated care.

A few studies have attempted to develop models to predict mortality and disease severity based on a large array of clinical variables associated with COVID-19 infection 10-14. Most of these prediction studies investigated patients from China, used logistic regression, and had small sample size and small number of clinical variables. Machine learning (ML) is increasingly being used in medicine, because of its ability to analyze large number of variables 15-17. ML uses computer algorithms to learn relationships amongst different data elements to inform outcomes without the need to explicitly specify the exact relationship, in contrast to conventional analysis methods. Many studies have shown that machine learning methods outperform humans in many tasks in medicine 18. With increasing computing power and growing relevance of big data in medicine, ML is expected to play an important role in clinical practice.

The goal of this study was to develop and compare different machine learning algorithms to predict the likelihood of ICU admission and mortality in COVID-19 patients. We identified the top few variables amongst the large array of clinical variables that were most predictive of the likelihood of ICU admission and mortality.

## Methods

### Patient population and data description

The retrospective study was approved by the Human Subjects Committee with an exemption for informed consent and HIPAA waiver. The data were collected from Electronic Medical Record and REDCAP database of the COVID-19 Patient under Investigation (PUI) registry of Stony Brook Hospital from March 9, 2020 to May 27, 2020. A subset of this dataset has been analyzed for a different purpose previously 19. The inclusion criteria were patients tested positive for severe acute respiratory syndrome coronavirus 2 (SARS-CoV-2) and admitted to the hospital. Exclusion criteria were patients younger than 21 years old, patients still in the hospital, and missing data.

**Figure 1:**
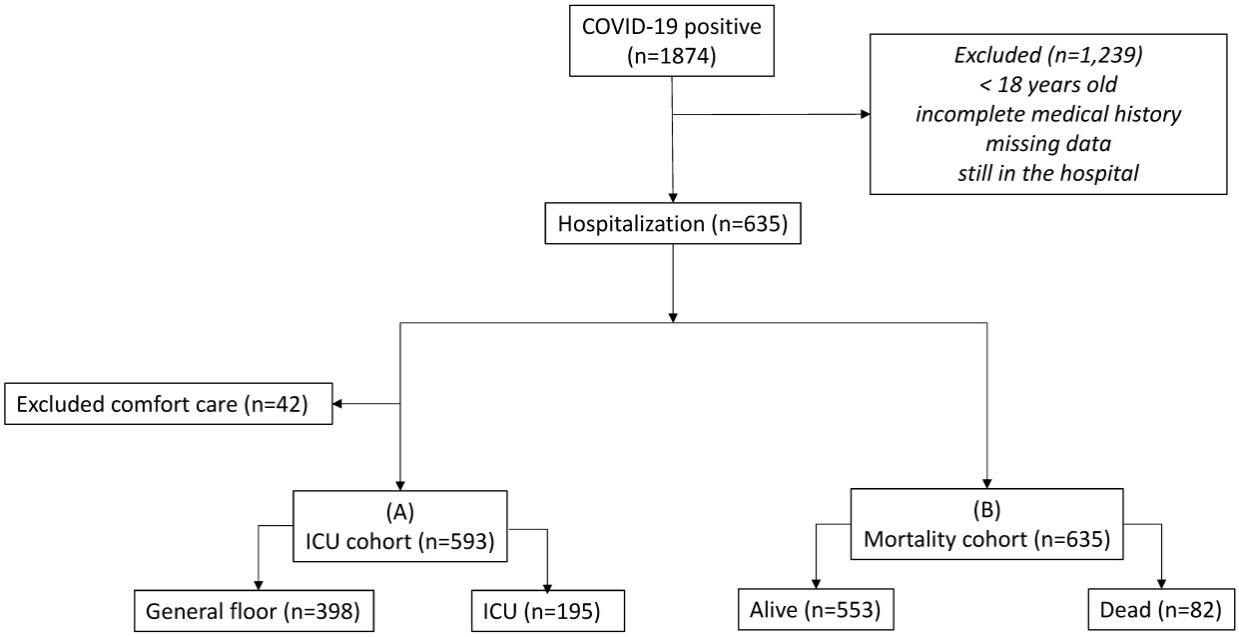
Flowchart of patient selection.

**Figure 1** shows the flowchart. There were 1874 patients tested positive for COVID-19. After applying the inclusion and exclusion criteria, 635 COVID-19 positive patients were used in our analysis. In the alive versus dead comparison, there were 553 alive and 82 dead. In the general floor versus ICU comparison, patients treated for comfort care were excluded (n=42) because these patients would have been sent to ICU if they were full code. There were 195 admitted to ICU and 398 to general floor. This ICU group included direct ICU admission and upgraded from general floor to ICU.

The input variables included demographic information (age, gender, ethnicity and race), chronic comorbidities (smoking, diabetes, hypertension, asthma, chronic obstructive pulmonary disease (COPD), coronary artery disease, heart failure, cancer, immunosuppression and chronic kidney disease), vital signs (heart rate, respiratory rate, pulse oxygen saturation (SpO2), systolic blood pressure and temperature), and laboratory tests at admission (alanine aminotransferase (ALT), brain natriuretic peptide, C-creative protein (CRP), D-dimer, ferritin, lactate dehydrogenase (LDH), leukocytes, lymphocytes, procalcitonin and troponin). Demographic information and chronic comorbidities were collected at admission to the Emergency Department.

### Machine learning model building

Two separate models were constructed for evaluation using different machine learning algorithms: 1) death vs discharged alive; 2) ICU vs general admission. Categorical predictors (e.g. ethnicity and race) were coded as dummy variables and continuous predictor (e.g. age, vital signs and laboratory tests) were standardized before machine learning. Multiple imputation with predictive mean matching method was used to impute missing values in vital signs and laboratory tests using the Multivariate Imputation by Chained Equations in R (statistical analysis software 4.0) 20. No imputation was performed for a predictor with missing more than 15%. Brain natriuretic peptide and troponin had more than 30% missing and were excluded from the analyses. As a result, 24 predictors were included as input features for machine learning models.

Logistic regression and four machine learning algorithms were considered for prediction models: random forest, Xgboost, kernel support vector machine (SVM) and neural network (packages “randomForest”, “xgboost”, “caret”, and “h2o” in R Statistical Analysis software, v4.0). Breiman's algorithm was performed for the random forest model. The number of trees to grow was set at a large number of 500. The minimum node size is set as 1 for the dichotomous classification and the maximum node size is not limited. In Xgboost models, gbtree was used for gradient boosting and the logistic regression objective function was used for classification. The learning rate parameter eta was set at 0.2 since the typical range for eta is from 0.01 to 0.3 as a lower value requires more computation and more iterations. The maximum number of iterations for gradient descent converge was set 100. Linear, polynomial and radial kernel were explored for SVM and the optimal kernel method was selected based on the prediction performance. As a result, the linear kernel achieved the highest AUC under ROC curves among the three kernel methods and were used for both mortality and ICU models. For neural network model, rectifier activation function for deep learning was used for classification. Two layers and ten nodes for each layer were set for the initiative model and the number of data iteration was set at 100 times.

Feature importance was determined using different methods in different machine learning algorithms. Random forest ranks predictors by mean decrease in Gini index 21. The corrected permutation approach was performed in the following two steps. First, the outcome was permuted 50 times and a nonparametric null distribution of feature importance was obtained, and second, the random forests with selected significant features were obtained. In Xgboost, mean decrease in Gini index and gain in the improvement in accuracy is used to evaluate the contribution of each predictor and neural network uses the Gedeon method for feature importance 22. To select top important predictors, 1000 rounds of permutation tests was performed. In each round, the original dataset was randomly split into training and testing sets with a ratio of 80%:20%. Predictors were then ranked by their importance in each of the four machine learning models. Percentages of times ranked on top 5 over 1000 permutation tests for each predictor were then calculated and used to determine the final rank of feature importance. Prediction performance was evaluated by area under the curve (AUC) of the receiver operating characteristic (ROC) curve for the test data set, sensitivity, and specificity. The average prediction performance was obtained with 1000 runs.

## Results

There were 635 COVID-19 positive hospitalized patients in our analysis. Descriptive statistics of demographics, chronic comorbidities, vital signs, and laboratory tests are presented. **Table 1** shows the comparison between the discharged alive versus dead group. Age, race, Hispanic were significantly different (p<0.05) between groups. Coronary artery disease, COPD, CKD, hypertension, heart failure, and smoking were significantly different between group (p<0.05), except asthma, carcinoma, diabetes and immunosuppression (p>0.05). CRP, D-dimer, LDH, leukocytes, lymphocytes, procalcitonin, SpO2, respiration rates and temperature were statistically different between groups (p<0.05), but not ALT, ferritin, heart rate, and SBP (p>0.05).

**Table 2** shows the comparison ICU vs general floor admission group. Patients treated for comfort care were excluded from this group (n=42) because these patients would have been sent to ICU if they were full code. Coronary artery disease, COPD, carcinoma, CKD, hypertension, heart failure, and smoking were significantly different between group (p<0.05), but not asthma, diabetes and immunosuppression (p>0.05). CRP, D-dimer, ferritin, LDH, leukocytes, lymphocytes, procalcitonin, SpO2, respiration rates, and temperature were statistically different between groups (p<0.05), but not ALT, heart rate, and SBP (p>0.05).

### Predictive performance

Predictive performance of each machine learning algorithms is shown in **Table 3**. The AUC of the mortality model ranged from 84% to 89%. The AUC of the ICU model ranged from 72% to 78%. Specificity was generally better than sensitivity. Random forest and Xgboost achieved better prediction AUC both in mortality and ICU models than the support vector machine and neural network. Random forest has the highest AUC for mortality (89%) and Xgboost has high AUC for ICU (79%).

### Feature importance

To evaluate the contribution of each predictor, predictors were ranked by their importance through 1000 the permutation tests. **Table 4** shows the feature ranking of all clinical variables based on individual AUCs and the permutation tests using logistic regression and machine learning algorithms. For all predictive models of mortality, the top common predictors were consistent across model and they were age, procalcitonin, C-creative protein, lactate dehydrogenase, and D-dimer. For all predictive models of ICU admission, the top common predictors were comparatively less consistent across models and they were procalcitonin, lactate dehydrogenase, C-creative protein, pulse oxygen saturation, ferritin and temperature. The common top predictors of mortality and ICU admission were procalcitonin, LDH, CRP, and SpO2.

## Discussion

This study investigated different machine learning algorithms to predict the likelihood of ICU admission and mortality in COVID-19 patients using clinical characteristics and laboratory results at admissions. The top 6 mortality predictors were age, procalcitonin, C-creative protein, lactate dehydrogenase, D-dimer and lymphocytes. The top 6 ICU admission predictors are procalcitonin, lactate dehydrogenase, C-creative protein, pulse oxygen saturation, ferritin and temperature. The best machine learning algorithms predicted mortality with 0.88 AUC and ICU admission with 0.79 AUC.

Most of the top predictors of mortality and ICU admission overlapped and they were procalcitonin, LDH, CRP, and SpO2. Procalcitonin is usually found to be elevated during bacterial infections, and less so during viral infection. Its elevation in critically ill COVID-19 patients could suggest the occurrence of potential bacterial co-infections or a decreased host immune response, both leading to worse outcome in these patients 23. LDH reflects tissue damage and has been known to be elevated in COVID-19 infection and other lung infections 2, 3. Elevated CRP, a blood marker of inflammation, suggests inflammatory response and tissue damage in the body 24. The surge of inflammation and the associated cytokine storm as a consequence have been associated with worse outcomes in COVID-19. Low SpO2 indicates failure of the lungs to oxygenate blood effectively, leading to hypoxia and respiratory failures that lead to mortality.

Some top predictors also differed for mortality and ICU admission. Age and D-dimer were uniquely associated with mortality whereas temperature and ferritin were uniquely associated with ICU admission. It is not surprising that old age is associated with a higher mortality. D-dimer, a small fragment protein by-product from breaking down blood clot, is indicative of a hyper-coagulability as a result of severe inflammatory reaction 25. These findings could explain why elevated D-dimer is associated with high mortality rate. On the other hand, high temperature might result in higher likelihood of ICU admission but not mortality. Similarly, elevated ferritin is a marker of aberrant iron metabolism that could render the lungs susceptible to oxidative damage 26. These findings suggest that abnormal temperature and ferritin are likely to cause severe disease requiring ICU care, but might be “treatable” or reversible as they are not significant predictors of mortality. Further research is needed to elucidate effective treatments.

It is also interesting to note that symptoms, comorbidities, and race/ethnicity were not amongst the 6 top predictors of mortality and ICU admission, although some of these variables have been associated with mortality or critical illness previously 27-30. Symptoms are subjective and highly variable, thus it is not surprising they were not highly ranked 31. A few studies have previously reported that patients with multiple comorbidities 27, 29 and certain race/ethnicity 28, 30 showed higher mortality rate or more likely to need escalated care. Comorbidity did not rank high on our cohort relative to other variables, likely because of the small sample sizes or that the clinical variables were indeed more predictive. Note that previous studies did not directly compare comorbidities and other clinical variables, and thus it is not known or not well established whether comorbidities are more predictive of mortality or of the need for escalated care relative to other clinical variables. Our sample sizes on the race and ethnicity cohorts were likely insufficient to reach meaningful conclusion. Further studies are warranted.

Another novelty of our study is that we performed analysis using 5 different predictive models. Random forests and Xgboost showed higher prediction accuracy than SVM and neural network. The random forests algorithms performed better than the neural networks likely due to small sample size. Overall, we found that all models predicted mortality better than ICU admission. Top common predictors were more consistent across different machine-learning models for predicting mortality than ICU admission. This is not unexpected as ICU admission decision were likely more variable because of how each physician practices and of how the pandemic has progressed temporally.

Although all these top predictors have been previously associated with COVID-19 infection 1-3, only a few studies have attempted to develop methods to predict mortality and disease severity. Lu et al. created a three-tiered risk score based on only two variables, age and CRP thresholds, to determine mortality 10, Xie et al. reported age, lymphocyte count, LDH and SpO_2_ to be independent predictors of mortality 11. Ji et al. predicted stable versus progressive COVID-19 patients based on whether their conditions worsened during hospitalization 12. They reported comorbidity, older age, lower lymphocyte and higher LDH at presentation to be independent high-risk factors for COVID-19 progression. A nomogram of these 4 factors yielded a concordance index of 0.86. Jiang et al. found mildly elevated alanine aminotransferase, myalgias, and hemoglobin at presentation to be predictive of severe acute respiratory distress syndrome (ARDS) of COVID-19 with 70% to 80% accuracy 14. However, this study had small and non-uniform clinical variables from different hospitals. Although some of the predictors of mortality were shared amongst these and our studies, there is currently no consensus as to which clinical variables are most predictive of mortality or the needs for escalated care. These differences in findings could be due to different outcome measures (mortality, ARDS, and disease severity), patient cohorts, different disease severity at admission, hospital environment, and analysis methods employed, among other factors. Our study differed from previously studies in several ways. We employed ML, in contrast to the majority of previous studies which used logistic regression. Our models identified top 6 predictors that accurately predicted both the needs for escalated care and mortality. We also compared different ML methods. Our study included comparatively large sample size and is amongst the few that described a patient cohort in the United States to date.

This study has several limitations. Although our study had a reasonably large sample size from a major academic hospital in New York, a temporal epicenter of the COVID-19 pandemic, it is a retrospective study carried out in a single hospital. These findings need to be replicated in large and multi-institutional settings for generalizability. We only analyzed clinical variables at admission. Longitudinal changes of these clinical variables need to be studied. It is important to note that the COVID-19 pandemic circumstance is unusual and evolving. ICU admission of COVID-19 patients may depend on individual hospital's patient load, practice, and available resources, which also differ amongst countries. Our institution to date has not been constrained by number of ICU beds nor mechanical ventilators in this study time frame. As in all observational studies, other residual confounders may exist that were not accounted for in our analysis. Future prospective studies validating our predictive models are warranted.

## Conclusion

We implemented and compared different machine learning algorithms to predict the likelihood of ICU admission and mortality in COVID-19 patients. This approach has the potential to provide frontline physicians with an objective tool to manage COVID-19 patients more effectively in time-sensitive, stressful and potentially resource-constrained environments.

### Key points

- ***Question:*** What are the top clinical parameters that predict ICU admission and mortality associated with COVID-19 infection?
- ***Findings:*** The top 6 mortality predictors were age, procalcitonin, C-creative protein, lactate dehydrogenase, D-dimer and lymphocytes. The top 6 ICU admission predictors are procalcitonin, lactate dehydrogenase, C-creative protein, pulse oxygen saturation, temperature and ferritin. The best machine learning algorithms predicted mortality with 0.88 AUC and ICU admission with 0.79 AUC.
- ***Meaning:*** This predictive model accurately predicts ICU admission and mortality in COVID-19 infection. It may prove useful for frontline physicians in clinical decision making.

## Figures and Tables

**Figure 2:**
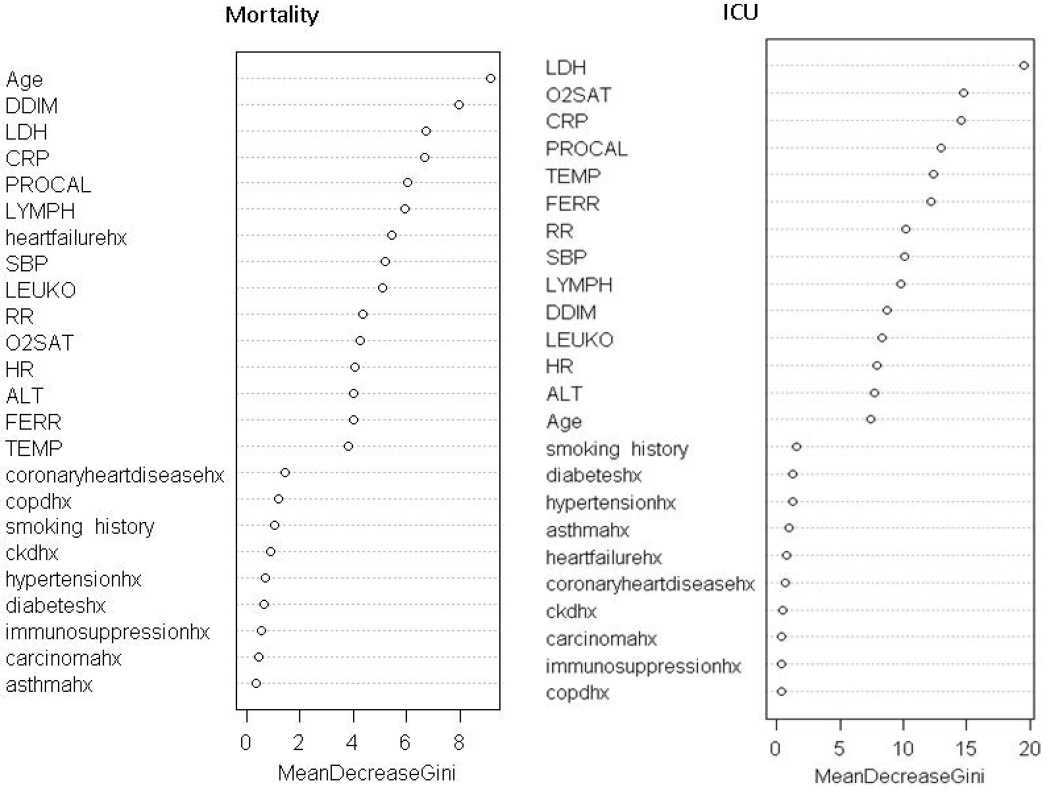
Feature rank of random forest models.

**Table 1:** Demographic, comorbidity, laboratory findings by survived and non-survived group

	Survived (n=553)	Non-Survived (n=82)	*P* value
**Demographics**			
Age, mean (std), y	57.71 (16.76)	73.62 (13.53)	<0.0001
Male	327 (59.1%)	53 (64.6%)	0.343
Race			0.002
Caucasian	251 (45.4%)	53 (64.6%)	
African American	38 (6.87%)	7 (8.5%)	
Other	264 (47.7%)	22 (26.8%)	
Hispanic	169 (30.6%)	8 (9.8%)	<0.0001
**Comorbidity**			
Asthma	38 (6.9%)	3 (3.7%)	0.269
Coronary artery disease	59 (10.7%)	25 (30.5%)	<0.0001
Chronic obstructive pulmonary disease	31 (5.6%)	15 (18.3%)	<0.0001
Carcinoma	32 (5.8%)	7 (8.5%)	0.333
Chronic kidney disease	37 (6.7%)	14 (17.1%)	0.001
Diabetes	148 (26.8%)	25 (30.5%)	0.48
Hypertension	243 (43.9%)	52 (63.4%)	0.001
Heart failure	12 (2.2%)	22 (26.8%)	<0.0001
Immunosuppression	31 (5.6%)	8 (9.8%)	0.144
Smoking	118 (21.3%)	36 (43.9%)	<0.0001
**Laboratory test and vital sign**			
Alanine aminotransferase, U/L	45.05 (48.22)	44.81 (54.07)	0.967
C-reactive protein, mg/L	10.22 (8.74)	15.69 (10.53)	<0.0001
D-dimer, nmol/L	835.89 (3519.6)	3037.9 (7692.1)	<0.0001
Ferritin, μg/L	1130.7 (1425.3)	1350.0 (1877.6)	0.255
Lactate dehydrogenase, U/L	381.24 (170.30)	489.38 (242.88)	<0.0001
Leukocytes×109 /liter	7.86 (4.21)	9.22 (5.41)	0.009
Lymphocytes %	15.35 (9.21)	11.72 (10.21)	0.001
Procalcitonin, ng/mL	0.80 (4.75)	4.18 (22.28)	0.002
Heart Rate, bpm	101.58 (66.72)	98.94 (21.39)	0.722
Pulse oxygen saturation, %	92.88 (7.01)	90.32 (8.63)	0.003
Respiratory rate, rate/min	21.95 (6.76)	24.67 (8.29)	0.001
SBP, mmHg	127.61 (24.43)	129.30 (29.83)	0.57
Temperature, °C	37.68 (0.90)	37.33 (0.78)	0.002

*P* values are based on Chi-square test or two-sample t-test.

**Table 2:** Demographic, comorbidity, laboratory findings by general floor and ICU group

	General floor (n=398)	ICU (n=195)	*P* value
**Demographics**			
Age, mean (std), y	57.68 (17.57)	59.70 (14.82)	0.168
Male	222 (55.8%)	136 (67.9%)	0.001
Race			0.080
Caucasian	190 (47.7%)	81 (41.5%)	
African American	32 (8%)	10 (5.1%)	
Other	176 (44.2%)	104 (53.3%)	
Hispanic	118 (29.6%)	56 (28.7%)	0.220
**Comorbidity**			
Asthma	25 (6.3%)	16 (8.2%)	0.386
Coronary artery disease	46 (11.6%)	22 (11.3%)	0.921
Chronic obstructive pulmonary disease	25 (6.3%)	11 (5.6%)	0.759
Carcinoma	25 (6.3%)	9 (4.6%)	0.412
Chronic kidney disease	28 (7.0%)	16 (8.2%)	0.610
Diabetes	104 (26.1%)	58 (29.7%)	0.354
Hypertension	170 (42.7%)	96 (49.2%)	0.134
Heart failure	10 (2.5%)	10 (5.1%)	0.097
Immunosuppression	22 (5.5%)	12 (6.2%)	0.758
Smoking	56 (14.1%)	49 (25.1%)	0.0009
**Laboratory test and vital sign**			
Alanine aminotransferase, U/L	44.01 (48.97)	48.19 (46.74)	0.321
C-reactive protein, mg/L	8.21 (7.54)	15.44 (10.29)	<0.0001
D-dimer, nmol/L	864.91 (3863.9)	939.58 (2103.2)	0.801
Ferritin, μg/L	882.11 (1275.8)	1469.2 (1401.1)	<0.0001
Lactate dehydrogenase, U/L	340.30 (148.89)	481.81 (191.99)	<0.0001
Leukocytes×109 /liter	7.57 (4.06)	8.73 (4.57)	0.002
Lymphocytes %	16.73 (9.57)	12.25 (8.37)	<0.0001
Procalcitonin, ng/mL	0.59 (2.61)	2.66 (15.76)	0.011
Heart Rate, bpm	99.23 (48.71)	107.09 (87.96)	0.163
Pulse oxygen saturation, %	94.43 (3.68)	88.92 (10.52)	<0.0001
Respiratory rate, rate/min	20.81 (5.78)	25.03 (8.45)	<0.0001
SBP, mmHg	128.58 (23.44)	126.46 (29.25)	0.342
Temperature, °C	37.80 (3.34)	37.75 (0.95)	0.839

*P* values are based on Chi-square test or two-sample t-test.

**Table 3:** Predictive performance of machine learning algorithms

Algorithms	Mortality			ICU		
	AUC (SD)	Sensitivity	Specificity	AUC (SD)	Sensitivity	Specificity
Random Forests	89.0% (1.3%)	76.4%	89.5%	78.1% (3.1%)	73.4%	79.6%
Xgboost	88.4% (1.9%)	30.3%	96.8%	78.9% (2.9%)	54.2%	86.0%
Kernel SVM	87.8% (4.3%)	20.8%	99.5%	76.1% (2.2%)	43.3%	92.3%
Neural network	84.4% (2.6%)	36.8%	90.3%	71.8% (4.4%)	56.7%	77.8%
Logistic	81.7%	26.2%	90.1%	66.9%	55.3%	78.5%

**Table 4:** Significant features of logistic regression (p<0.05) and top 6 features of machine learning algorithms for mortality and ICU models

Logistic	Random Forests	Xgboost	SVM	Neural Network
**Mortality**				
Age	Age	Age	Age	CKD
Heart Failure	D-Dimer	D-Dimer	D-Dimer	Ferritin
LDH	CRP	COPD	Procalcitonin	Age
CRP	LDH	CRP	CRP	Respiratory Rate
Hypertension	Procalcitonin	Procalcitonin	Lymphocytes	ALT
Immunosuppression	Lymphocytes	LDH	LDH	LDH
**ICU**				
CRP	Procalcitonin	LDH	Procalcitonin	SpO_2_
LDH	LDH	Procalcitonin	LDH	Heart Rate
SpO_2_	CRP	CRP	CRP	Age
Heart Failure	SpO_2_	SpO_2_	Ferritin	Ferritin
Smoking	Temperature	Temperature	SpO_2_	LDH
SBP	Ferritin	Ferritin	lymphocytes	CRP

ALT: Alanine aminotransferase, LDH: lactate dehydrogenase, CRP: C-reactive protein, SBP: systolic blood pressure. COPD: chronic obstructive pulmonary.
